# Evolution of the *Plasmodium vivax* multidrug resistance 1 gene in the Greater Mekong Subregion during malaria elimination

**DOI:** 10.1186/s13071-020-3934-5

**Published:** 2020-02-12

**Authors:** Huguette Gaelle Ngassa Mbenda, Meilian Wang, Jian Guo, Faiza Amber Siddiqui, Yue Hu, Zhaoqing Yang, Veerayuth Kittichai, Jetsumon Sattabongkot, Yaming Cao, Lubin Jiang, Liwang Cui

**Affiliations:** 1 Division of Infectious Diseases and International Medicine, Department of Internal Medicine, Morsani College of Medicine, University of South Florida, Tampa, FL USA; 2 Department of Immunology, College of Basic Medical Sciences, China Medical University, Shenyang, 110001 China; 3 Department of Laboratory Medicine, Shanghai East Hospital, Tongji School of Medicine, Shanghai, China; 4 Department of Pathogen Biology and Immunology, Kunming Medical University, Kunming, Yunnan China; 5 Mahidol Vivax Research Unit, Faculty of Tropical Medicine, Mahidol University, Bangkok, Thailand; 6 Unit of Human Parasite Molecular and Cell Biology, Key Laboratory of Molecular Virology and Immunology, Institut Pasteur of Shanghai, Chinese Academy of Sciences, Shanghai, China

**Keywords:** *Plasmodium vivax*, Multidrug resistance 1 gene, Chloroquine resistance, Genetic diversity, Selection

## Abstract

**Background:**

The malaria elimination plan of the Greater Mekong Subregion (GMS) is jeopardized by the increasing number of *Plasmodium vivax* infections and emergence of parasite strains with reduced susceptibility to the frontline drug treatment chloroquine/primaquine. This study aimed to determine the evolution of the *P. vivax multidrug resistance 1* (*Pvmdr1*) gene in *P. vivax* parasites isolated from the China–Myanmar border area during the major phase of elimination.

**Methods:**

Clinical isolates were collected from 275 *P. vivax* patients in 2008, 2012–2013 and 2015 in the China–Myanmar border area and from 55 patients in central China. Comparison was made with parasites from three border regions of Thailand.

**Results:**

Overall, genetic diversity of the *Pvmdr1* was relatively high in all border regions, and over the seven years in the China–Myanmar border, though slight temporal fluctuation was observed. Single nucleotide polymorphisms previously implicated in reduced chloroquine sensitivity were detected. In particular, M908L approached fixation in the China–Myanmar border area. The Y976F mutation sharply decreased from 18.5% in 2008 to 1.5% in 2012–2013 and disappeared in 2015, whereas F1076L steadily increased from 33.3% in 2008 to 77.8% in 2015. While neutrality tests suggested the action of purifying selection on the *pvmdr1* gene, several likelihood-based algorithms detected positive as well as purifying selections operating on specific amino acids including M908L, T958M and F1076L. Fixation and selection of the nonsynonymous mutations are differently distributed across the three border regions and central China. Comparison with the global *P. vivax* populations clearly indicated clustering of haplotypes according to geographic locations. It is noteworthy that the temperate-zone parasites from central China were completely separated from the parasites from other parts of the GMS.

**Conclusions:**

This study showed that *P. vivax* populations in the China–Myanmar border has experienced major changes in the Pvmdr1 residues proposed to be associated with chloroquine resistance, suggesting that drug selection may play an important role in the evolution of this gene in the parasite populations.
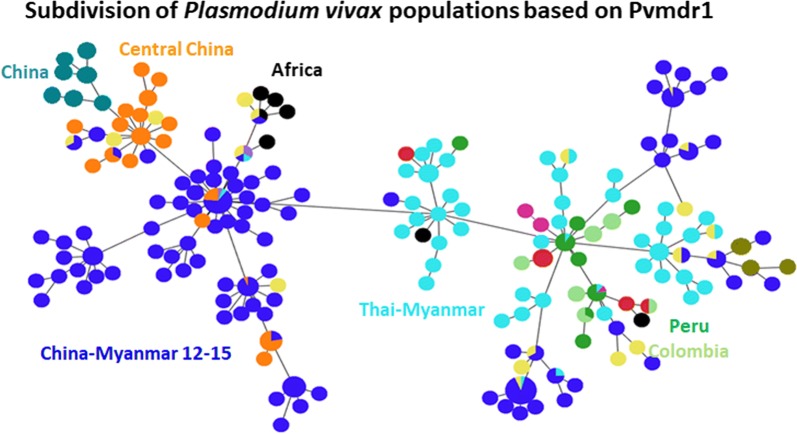

## Background

Worldwide, around 100 million cases of *Plasmodium vivax* infections are registered annually with majority of them occurring in the Asian Pacific Region [1, 2]. Most countries in Southeast Asia are making steady progress in reducing the malaria burden; the six countries in the Greater Mekong Subregion (GMS) have set their goals to eliminate malaria by 2030 [1, 3]. This elimination plan is, however, challenged by the difficulties to eliminate *P. vivax* because of its several biological features such as very low blood parasitemia that is often missed by conventional detection methods, and formation of hypnozoites in the liver of an infected individual that are responsible for subsequent relapses [4]. Despite increased control efforts in the GMS, *P. vivax* transmission along international borders remains high [5, 6].

Understanding the genetics of drug resistance in *P. vivax* is important for implementing an effective chemotherapeutic strategy and monitoring the progress of elimination [7]. Whilst the mechanisms of drug resistance in *Plasmodium falciparum* are much better understood, those in *P. vivax* are largely unknown. Chloroquine (CQ) has been withdrawn from treating *P. falciparum* malaria in most endemic countries due to widespread resistance to this drug [8, 9], but CQ-primaquine (PQ) combination is still the first-line treatment for *P. vivax* infections in most endemic countries [10, 11]. Unfortunately, there is an increased number of reports of reduced susceptibility of *P. vivax* parasites to CQ from malaria-endemic areas, including the GMS countries [2, 8, 12–21]. Despite this, there is still a lack of a confirmed marker(s) for CQ resistance in *P. vivax.* Several studies have indicated that mutations in the *multidrug resistant 1* gene (*Pvmdr1*) may be used as markers for CQ resistance surveillance [22, 23]. *In vitro* drug susceptibility assays identified an association between higher copy numbers of the *Pvmdr1* and increased CQ IC_50_ values [24, 25], although the cut-off IC_50_ value for CQ resistance is uncertain. More recently, a connection has been made between the copy number of the *Pvmdr1* harboring the Y976F/F1076L mutations and treatment failure in severe *P. vivax* malaria cases [26, 27]. In addition, the M908L and T958M mutations were shown to be associated with reduced *in vitro* CQ sensitivity [28]. However, some studies failed to detect a link between the *Pvmdr1* mutations and reduced CQ sensitivity, raising doubts on the suitability of the *Pvmdr1* mutations as markers for CQ resistance [29, 30].

Population genomics studies revealed great diversity of the *P. vivax* parasites as compared to *P. falciparum* [31, 32], indicating more stable populations. Moreover, signals of natural selection have been detected in *P. vivax*, highlighting the ability of *P. vivax* to evolve in response to antimalarial drug pressure and changing environments in the human host as well as in the mosquito vector [32]. For instance, dihydropteroate synthase and dihydrofolate reductase genes that are associated with resistance to antifolate drugs were found to be selected in *P. vivax* [31]. In the GMS, *P. vivax* parasites were found to exhibit high levels of genetic diversity in Thailand [33], southern China, and Myanmar [34]. In this study, we focused on the genetic diversity of the *Pvmdr1* gene in the vivax-endemic area along the China–Myanmar border, hoping to understand the evolution of the parasites amid the falling CQ treatment efficacy [35] and increased proportions of vivax malaria in most areas of the GMS [3].

## Methods

### Study sites and samples

Clinical *P. vivax* samples were collected from 330 patients with acute *P. vivax* malaria attending different malaria clinics. Among them, 39 and 16 were from Anhui Province of central China in 2004 and 2006–2008, respectively. For the longitudinal samples from the China–Myanmar border, 27, 129 and 119 samples were collected in 2008, 2012–2013 and 2015, respectively, giving a total of 275 samples from this border region. Finger-prick blood samples of microscopy-confirmed *P. vivax* cases were spotted onto Whatman 3M filter papers.

### Sequencing of the *Pvmdr1* gene

Genomic DNA was extracted from dried blood spots on filter paper using the QIAamp DNA Mini kit (Qiagen, Hilden, Germany) according to the manufacturer’s protocol. Genotyping of two polymorphic genes (*msp3α* and *msp3β*) by PCR/RFLP was done to distinguish single from mixed-strain infections [36, 37]. For PCR amplification of the *Pvmdr1* gene, primary PCR was performed using primers P1F and P1R and two fragments were amplified by semi-nested PCR with primer pairs P1F × N-PR and N-PF × P1R, respectively (Additional file 1: Table S1). PCR was performed using the Advantage 2 polymerase mix (Takara Bio, Mountain View, USA) and PCR products were sequenced in both directions using the Sanger method on an ABI DNA analyzer. The *Pvmdr1* sequences were assembled and edited using DNAStar (Lasergene, Madison, USA). The *Pvmdr1* sequences generated from this study are available in GenBank with the accession numbers: MN891946–MN891972; MN891973–MN892091; MN892092–MN892220; MN892221–MN892236; and MN892237–MN892275. In addition, 98 *Pvmdr1* sequences from parasites collected in western (Tak and Kanchanaburi provinces) and eastern Thailand (Ubon Rachathani Province) were also used for analysis [33]. All sequences were aligned with the reference *Pvmdr1* sequence from the Salvador I strain (PVX_080100) using Clustal Muscle 3.8 [38] incorporated in the MEGA7 software [39].

### Assessment of genetic diversity

All the *Pvmdr1* sequences were scanned for the presence of single nucleotide polymorphisms (SNPs). The genetic diversity of the *Pvmdr1* gene was assessed using DnaSP software v6.10 [40]. Haplotype diversity (Hd) of the *Pvmdr1* gene was estimated based on the number and frequency of the haplotypes, while nucleotide diversity was measured using two parameters: π, the average number of pairwise nucleotide differences per site [41] and θw, the number of segregating sites.

### Tests for detecting selection

To determine whether natural selection played a role in the evolution of *Pvmdr1*, we first performed a series of frequency-based tests including Tajima’s *D* test [42], Fu and Li’s F test [43], and Fu and Li’s D test [43] using the DnaSP v6.10 software. The Tajima’s *D* statistic calculates the normalized differences between the two measures of nucleotide diversity θw and π [42]. Both the Fu and Li’s D and F statistics rely on the difference between the number of polymorphic sites in external branches (polymorphisms unique to an extant sequence) and the number of polymorphic sites in internal phylogenetic branches (polymorphisms shared by extant sequences) [44]. For all statistical analyses, a *P*-value of ≤ 0.05 was considered significant. We also used McDonald–Kreitman (MK) test to examine departure from neutrality using *Plasmodium knowlesi mdr1* sequence as the outgroup [45]. The MK test compares the ratio of nonsynonymous to synonymous polymorphism within a species (Pn/Ps) and the ratio of nonsynonymous to synonymous substitutions between closely related species (dN/dS). Fisher’s exact test was used to assess statistical significance.

We then determined the nucleotide substitutions and the ratio of nonsynonymous (dN) to synonymous (dS) substitutions per site (dN/dS), using the Nei-Gojobori method [39] after Jukes-Cantor correction for multiple substitutions. Under the neutral model of evolution, dS is expected to be equal to dN. An excess of nonsynonymous substitutions (dN > dS) can be interpreted as positive selection [46], indicating that replacement substitutions increase fitness of the parasite, whereas a rarity of replacement changes (dN < dS) specifies that purifying selection might be working to remove such substitutions from the gene pool [47]. Statistical significance of the difference was estimated using the codon-based *Z*-test of selection in MEGA7 [39].

Finally, since selection is often directed at a few amino acids of a gene and sometimes can be camouflaged by purifying selection also acting on the gene [48], we conducted maximum likelihood tests in the HyPhy package implemented in the Data Monkey Web Server [39] to determine the specific amino acids targeted by selection [47]. Significant recombination events were tested in the DnaSP program and by genetic algorithm for recombination detection [49] incorporated in the Data Monkey Web Server before running the tests of selection.

### Prediction of possible effects of the Pvmdr1 mutations on protein function

To predict if any of the *Pvmdr1* mutations might affect the protein structure and function, we mapped these residues on a modeled 3D structure using the Sal I reference sequence. The homology model of PvMDR1 was built based on the structures of the multidrug transporter P-glycoprotein (Pgp) from *Caenorhabditis elegans* (4F4C) and mouse (4M1M and 3G61) using the multiple threading alignment in I-TASSER [50]. A confidence score (C-score) for estimating the quality of predicted models by I-TASSER was calculated [50]. Web-based software PROVEAN and SIFT (Sorting Intolerant from Tolerant) were utilized to predict the effect of amino acid mutations in PvMDR1 [51]. Mutations predicted to be deleterious according to both the software were mapped on the predicted 3D structure of PvMDR1.

### Population differentiation and linkage disequilibrium (LD)

To determine the genetic interrelationships among all parasite isolates, a phylogenetic tree was constructed using the Maximum Likelihood algorithm with 1000 bootstraps as implemented in MEGA7. The Sal I reference strain was represented as the wild type. In addition to the 275 *Pvmdr1* sequences obtained from this study, a total of 180 complete or nearly complete *Pvmdr1* sequences retrieved from GenBank and PlasmoDB (plasmodb.org) representing parasite isolates from 11 countries were also analyzed: 6 from China; 98 from Thailand; 5 from Papua New Guinea (PNG); 7 from Madagascar; 14 from Mexico; 20 from Colombia; 24 from Peru; 3 from Brazil; and one each from India, North Korea, and Mauritania. Each sequence was trimmed to remove low-quality segments, yielding 4137 bp of the 4395 bp *Pvmdr1* open reading frame. To estimate the proportion of genetic variance of the *Pvmdr1* gene due to population subdivision, Wright’s fixation index of inter-population variance in allele frequencies (*F*_ST_) was calculated. Pairwise linkage LD was used to determine the degree of random association between different mutations within this gene. The correlation coefficient (*R*^2^) between paired alleles was estimated using DnaSP v6.10 and the significance of each association was determined using the Fisher and Chi-square tests after Bonferroni correction.

### Haplotype network analysis

To visualize the distribution of the *Pvmdr1* polymorphisms across different *P. vivax* populations, haplotypes were constructed from nonsynonymous SNPs that were observed in more than two isolates. A minimum spanning tree was drawn using the median-joining algorithm in the PHYLOViZ software (https://www.phyloviz.net).

## Results

### Genetic diversity of the *Pvmdr1* gene

Mutations in the *Pvmdr1* gene have been associated with CQ resistance in *P. vivax* in some endemic areas. In light of the deteriorating CQ clinical efficacy for treating vivax malaria at the China–Myanmar border [35], we followed the evolution of the *Pvmdr1* gene in parasite populations from this region over a seven-year period and sequenced the full-length *Pvmdr1* gene in 275 *P. vivax* clinical samples. To put this study in context with parasites from other regions in the GMS, we also analyzed 98 *Pvmdr1* sequences from the western and eastern borders of Thailand [33]. For the longitudinal *P. vivax* clinical samples from the China–Myanmar border, 27 isolates collected in 2008 harbored 22 SNPs, of which 20 are nonsynonymous. In the 129 samples collected in 2012–2013, eight synonymous and 15 nonsynonymous SNPs were identified. In the 119 samples collected in 2015, 34 synonymous and 59 nonsynonymous SNPs were found. For the 55 temperate-zone *P. viv*ax isolates collected from central China in 2004–2008, 24 SNPs were found, of which 17 were nonsynonymous. Several of the nonsynonymous mutations in the *Pvmdr1* gene had allele frequencies of at least 5% (Table 1); eight were common in the China–Myanmar border populations from the three time points: S513R, G698S, L845F, A861E, M908L, T958M, F1076L and K1393N (Table 1). Among them, G698S, M908L, and T958M reached or almost reached fixation (97–100%). Six of the eight mutations were also detected in the central China parasite population with allele frequencies of ≥ 5% (Table 1). For the three SNPs (T958M, Y976F and F1076L) proposed to be associated with CQ resistance [23, 52], F1076L was fixed in the central China population. In the China–Myanmar border parasite populations, the prevalence of F1076L continually increased over time, from 33.3% in 2008 to 41.7% in 2012–2013 and 77.8% in 2015. In contrast, the Y976F mutation was not present in the central China parasite population, and its frequency in the border parasite populations was moderately high at 18.5% in 2008, but sharply decreased to 1.5% in 2012–2013 and was completely absent in the 2015 samples (Table 1).

**Table 1:** Frequency (%) of nonsynonymous mutations in *Pvmdr1* in the China–Myanmar border area and central China

Amino acid^a^	China–Myanmar border			Central China (*n* = 55)
	2008 (*n* = 27)	2012–2013 (*n* = 129)	2015 (*n* = 119)	
P8L	0	0	0	17.9
T409M	0	9.3	13.7	0
S513R	55.5	46.5	11.1	0
G520D	0	13.9	8.5	48.8
G698S	88.9	97.7	100	100
L845F	14.8	3.1	2.6	52.7
A861E	14.8	24.8	26.5	29.1
M908L	100	99.2	98.3	100
V945G	0	0	13.7	0
T958M	100	100	100	100
Y976F	18.5	1.5	0	0
F1076L	33.3	41.7	77.8	100
K1393N	29.6	45.7	16.2	0
S1450L	0	21.8	13.8	0

^a^The mutations listed are those having a MAF > 5% in at least one population

Overall, the genetic diversity of the *Pvmdr1* gene in the China–Myanmar border parasite populations was relatively high (π = 0.0009–0.0012) with slight fluctuation over the years. Similarly, haplotype diversity was also high in the China–Myanmar border parasite populations: 21, 33 and 75 haplotypes were identified in the 2008, 2012–2013 and 2015 samples, respectively (Table 2). Compared with the China–Myanmar border parasite populations, the genetic diversity of the *Pvmdr1* gene in parasites from central China was much lower (π = 0.0006). Yet, haplotype diversity of these temperate-zone parasites was high (0.914 ± 0.026). Except for the population of 2012–2013, the π value was lower than the θw value for the rest of populations, suggesting that most SNPs in the *Pvmdr1* gene were rare alleles.

**Table 2:** Diversity and neutrality tests of the *Pvmdr1* gene in *P. vivax* populations from different areas of the GMS

Populations	SNP		No. of haplotypes	Haplotype diversity (Hd ± SD)	Nucleotide diversity		Neutrality tests			Rm
	S	NS			θw	π	*D*	D*	F*	
China–Myanmar 2008 (*n* = 27)	2	20	21	0.983 ± 0.014	0.0012	0.0010	− 0.7839	− 1.2499	− 1.2950	4
China–Myanmar 2012–2013 (*n* = 129)	8	15	33	0.869 ± 0.023	0.0009	0.0009	0.0316	0.0357	0.0404	6
China–Myanmar 2015 (*n* = 119)	34	59	75	0.971 ± 0.008	0.0039	0.0012	− 2.2310**	− 4.2879**	− 4.0776**	15
Central China (*n* = 55)	7	17	23	0.914 ± 0.026	0.0010	0.0006	− 1.1179	− 2.1131	− 2.0953	3
Thailand–Myanmar (*n* = 69)^a^	10	21	44	0.974 ± 0.009	0.0015	0.0009	− 1.3670	− 2.7450*	− 2.6640*	5
Thailand–Cambodia (*n* = 29)^a^	7	7	15	0.926 ± 0.026	0.0008	0.0007	− 0.4540	− 0.9490	− 0.9320	4

^a^Analysis has been done in these populations on a near full-length *Pvmdr1* gene (4264 bp)

*Abbreviations*: S, synonymous; NS, nonsynonymous; SD, standard deviation; *D*, Tajima’s *D*; D*, Fu & Li’s D*, F*: Fu & Li’s F*, Rm, minimum recombination events

*P < 0.05, **P < 0.02

Compared with *P. vivax* parasite populations from other parts of the GMS, haplotype diversity in the China–Myanmar border in 2015 was similarly high (0.971 ± 0.008) as that from the Thailand–Myanmar border (0.974 ± 0.009) (Table 2). For all parasite populations from the three border areas (China–Myanmar, Thailand–Myanmar, and Thailand–Cambodia) in this study, the π value was lower than the θw value (Table 2), suggesting the prevalence of rare alleles as mentioned above.

### Mutations within the putative 3D model of PvMDR1

**Fig. 1:**
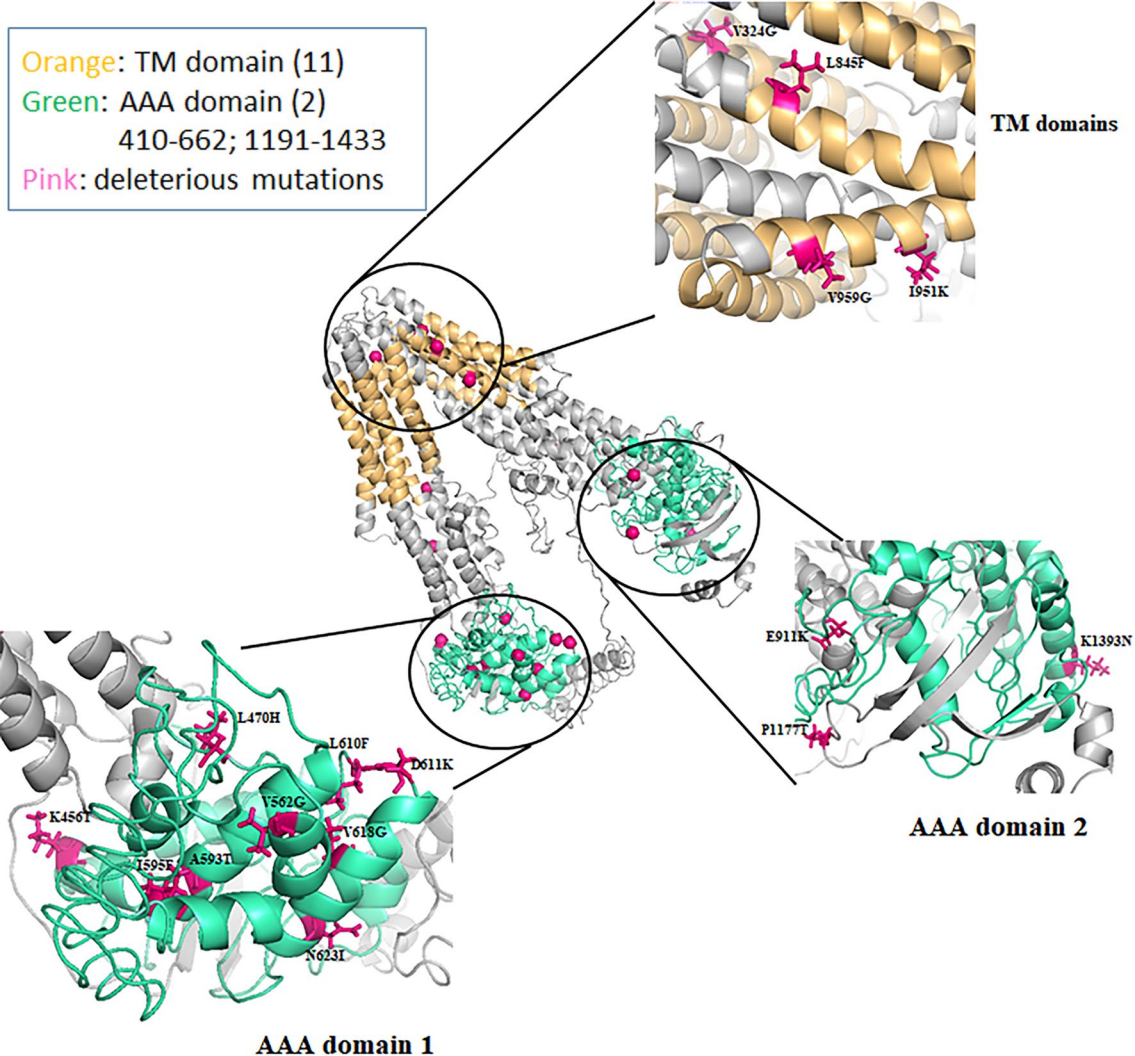
Mapping of the mutated residues predicted deleterious by PROVEAN and SIFT analyses. All the mutated residues that are predicted to be deleterious according to both Provean and SIFT analyses were mapped on the predicted 3D model structure of Pvmdr1

PvMDR1 is a member of the ATP-binding cassette (ABC) protein superfamily with two symmetric domains. Each domain has a transmembrane domain (TMD), consisting of three external loops and two internal helices that link six TMDs followed by a nucleotide binding domain (NBD) [53]. According to the protein alignment and domain mapping analyses, the two TMDs contain 5–6 transmembrane helices (at amino acids 62–84, 99–121, 171–193, 197–216, 281–303, 323–345, 825–847, 867–889, 940–962, 966–985 and 1062–1084), while the two NBDs, also referred to as the AAA domains, are located at amino acids 410–662 and 1191–1433, respectively (Fig. 1). The predicted domains in PvMDR1 show high sequence homology to the corresponding PfMDR1 functional domains [54]. For the 71 mutations reported in this study, 38 are predicted to be deleterious by at least one of the prediction programs (Additional file 2: Table S2), whereas 19 are predicted to be detrimental according to both Provean and SIFT analysis (Additional file 3: Table S3). To predict the effect of these 19 mutations on protein structure, we mapped the mutated residues on the predicted tertiary structure. The homology model of PvMDR1 built using I-TASSER aligned well with the *C. elegans* multidrug transporter P-glycoprotein. Except I595, 18 of the 19 amino acids are conserved in PfMDR1 protein sequence, indicating functional conservation and significance.

Of these 19 predicted deleterious mutations, V324G is located in the first TMD, whereas three (L845F, I951K, and V959G) are in the second TMD (Fig. 1). Notably, I951K represents a drastic change from a hydrophobic to a positively charged residue, which may disrupt the integrity of the TMD. Five mutations (Y348D, Y359D, E911K, D932N and P1177T) are located in the predicted inter-domain regions. Interestingly, most of the mutations with predicted adverse effects lie in the first NBD/AAA domain (K456T, L470H, V562G, A593T, I595F, L610F, D611K, V618G, N623I), whereas only one mutation (K1393N) is present in the second AAA domain. All these mutations can potentially affect the structural integrity of the protein by altering the charges, hydrophobicity, or size of the amino acids. In addition, the residues corresponding to L470, L610, and D611 in PfMDR1 are predicted to be involved in NDB dimerization [54], and mutations at these positions may hinder dimerization of the protein. Insights into the ATP-binding pockets can help decipher if any of these residues are involved in direct binding to ATP or some antimalarial drugs, allowing the determination of their potential role in transport and resistance.

### Departure from neutrality

Frequency-based neutrality tests were used to assess the evolution of the *Pvmdr1* gene. All the neutrality tests yielded negative values for all the sample sets except the 2012–2013 parasite population from the China–Myanmar border (Table 2). However, only the 2015 parasites from the China–Myanmar border significantly deviated from neutral with an excess of low-frequency polymorphisms, suggesting that the *Pvmdr1* gene in China–Myanmar border possibly experienced either a directional selection or population expansion during the seven years.

The dN-dS statistic generated by the *Z*-test was negative in all the populations, suggestive of purifying selection on *Pvmdr1*, albeit it was not statistically significant (Table 3). The MK test also indicated that the *Pvmdr1* gene was evolving under purifying selection in the central China and the China–Myanmar border populations. The number of recombination events was found to be very high in the 2015 border parasite population (Rm = 15), corroborating the high diversity observed in this population (Table 2).

**Table 3:** Tests for selection in *Pvmdr1* genes from parasite populations from the China–Myanmar border and central China

Populations	*Z*-test (dN-dS)	MacDonald & Kreitman test	
		Alpha-value	G-value
China–Myanmar border 2008 (*n* = 27)	− 1.094	− 0.554	0.976
China–Myanmar border 2012–2013 (*n* = 129)	− 0.850	− 2.003*	20.580***
China–Myanmar border 2015 (*n* = 119)	− 1.481	− 1.800***	21.470***
Central China 2004–2008 (*n* = 55)	− 0.304	− 1.566	4.262*

*P < 0.05, ***P < 0.001

The likelihood-based algorithms (SLAC [55], FEL [55], and FUBAR [56] implemented in the Datamonkey webserver [57]) all revealed positive as well as purifying selection at specific codons (Table 4). The number of sites negatively selected increased with the years in the China–Myanmar border populations. Among the mutations presumptively associated with CQ resistance, F1076L seemed to be positively selected in the 2008 and 2012–2013 China–Myanmar border populations, whereas M908L was positively selected in the 2015 population (Table 4). In comparison, none of these mutations were selected in the central China population (Table 4). It is noteworthy that of the two mutations M908L and T958M associated with reduced *in vitro* CQ sensitivity [28] and also highly prevalent in Thailand [33], M908L was positively selected in the China–Myanmar border parasites but not in the Thailand–Myanmar or the Thailand–Cambodia border parasite populations (Table 4). Furthermore, most of mutations with frequencies of ≥ 5% such as A861E, L845F, and K1393N (Table 1) were also selected in the China–Myanmar parasites (Table 4). In the Thailand–Cambodia border population, only F1076L was found to be positively selected (Table 4), whereas in the Thailand–Myanmar border populations, S513R, G698S, A861E, F1076L and K1393N were positively selected (Table 4).

**Table 4:** Codon-based tests for selection on *Pvmdr1* gene in parasite populations at various sites of the GMS

Populations	FEL	SLAC	FUBAR
China–Myanmar 2008 (*n* = 27)	Positively selected: 513, 1076	Positively selected: none	Positively selected: 513, 845, 1076, 1393
	Negatively selected: 44, 399, 529, 756, 1358, 1396	Negatively selected: 44, 529, 1358	Negatively selected: 44, 181, 399, 529, 687, 756, 1358, 1396
China–Myanmar 2012–2013 (*n* = 129)	Positively selected: 513	Positively selected: none	Positively selected: 513, 698, 845, 861, 976, 1076, 1393, 1450
	Negatively selected: 44, 493, 529, 1348, 1396	Negatively selected: 44, 529	Negatively selected: 44, 493, 529, 1348, 1396
China–Myanmar 2015 (*n* = 119)	Positively selected: 861, 908, 926	Positively selected: none	Positively selected: 231, 359, 409, 513, 562, 599, 612, 618, 838, 861, 896, 908, 926, 936, 951, 959, 1219, 1393, 1450
	Negatively selected: 23, 44, 214, 220, 298, 322, 347, 493, 501, 529, 554, 555, 574, 585, 587, 865, 910, 917, 939, 1004, 1190, 1233, 1238, 1358	Negatively selected: 44, 529, 322, 555, 574, 910, 1358	Negatively selected: 23, 44, 214, 220, 298, 322, 347, 493, 501, 529, 554, 555, 574, 585, 587, 865, 910, 917, 925, 939, 1004, 1067, 1190, 1233, 1238, 1348, 1358
Central China 2004–2008 (*n* = 55)	Positively selected: none	Positively selected: none	Positively selected: 8, 520, 845, 1233, 1390
	Negatively selected: 44, 687	Negatively selected: 1464	Negatively selected: 44, 75, 248, 529, 687, 1390
Thailand–Myanmar border (n = 69)	Positively selected: 698	Positively selected: 698	Positively selected: 513, 698, 861, 976, 1076, 1393
	Negatively selected: 44, 529, 1396	Negatively selected: 44, 172, 493, 529, 913, 1396	Negatively selected: 44, 172, 493, 529, 913, 1396
Thailand–Cambodia border (*n* = 29)	Positively selected: none	Positively selected: none	Positively selected: 1076
	Negatively selected: 44, 312, 529	Negatively selected: 44, 312	Negatively selected: 44, 312, 529, 908

The Hudson and Kaplanʼs lower bound on the minimal number of recombination events in an infinite site model computed with DnaSP revealed 4, 6, 15, and 3 minimum recombination events in the *Pvmdr1* gene from the China–Myanmar border populations in 2008, 2012–2013, and 2015 and the central China population, respectively (Table 2). In the Thailand–Myanmar and Thailand–Cambodia border regions, the minimum recombination events were 5 and 4, respectively (Table 2). On the other hand, analysis by using GARD (genetic algorithm for recombination detection) [49], a model-based approach that searches for putative breakpoints delimiting sequence regions having distinct phylogenies, found evidence of a breakpoint only in the Thailand–Myanmar border parasite population. The low number of recombination events in Thailand–Myanmar and Thailand–Cambodia border populations might be due to a recent expansion in effective parasite population. Focusing on the China–Myanmar border parasites, our study detected an increasing number of minimum recombination events from 2008 to 2015, highlighting a possible reduction of *P. vivax* effective population size during these years.

### Global distribution of the *Pvmdr1* haplotypes and geographical differentiation

**Fig. 2:**
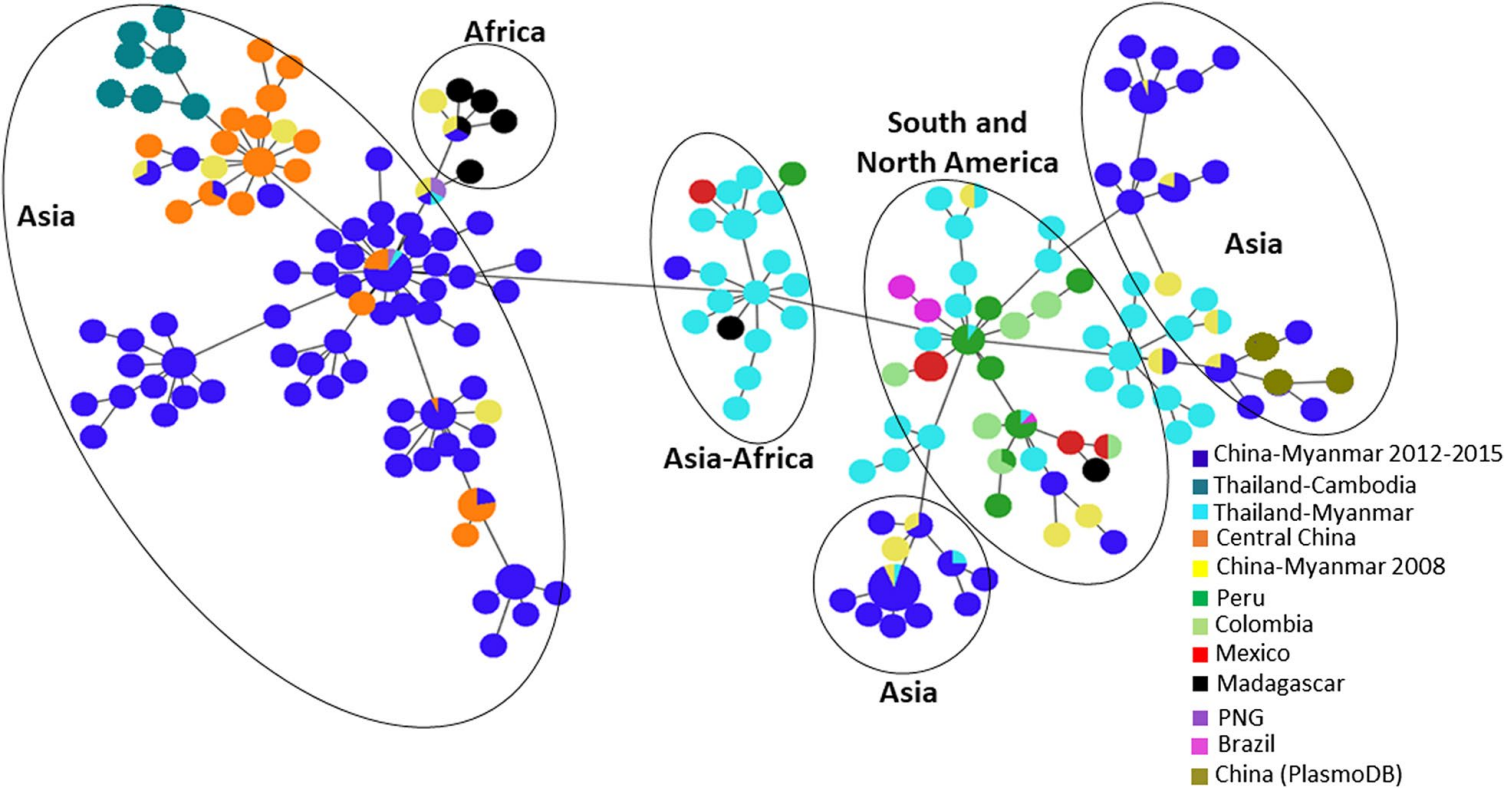
Minimum spanning network for global *P. vivax* parasite populations. The size of the pies reflects the frequency of a particular haplotype. The lengths of the lines connecting the pies, measured from their centers, are in proportion to the number of base pair substitutions separating the haplotypes. Color represents different countries. Haplotypes observed in different continents are encircled

**Fig. 3:**
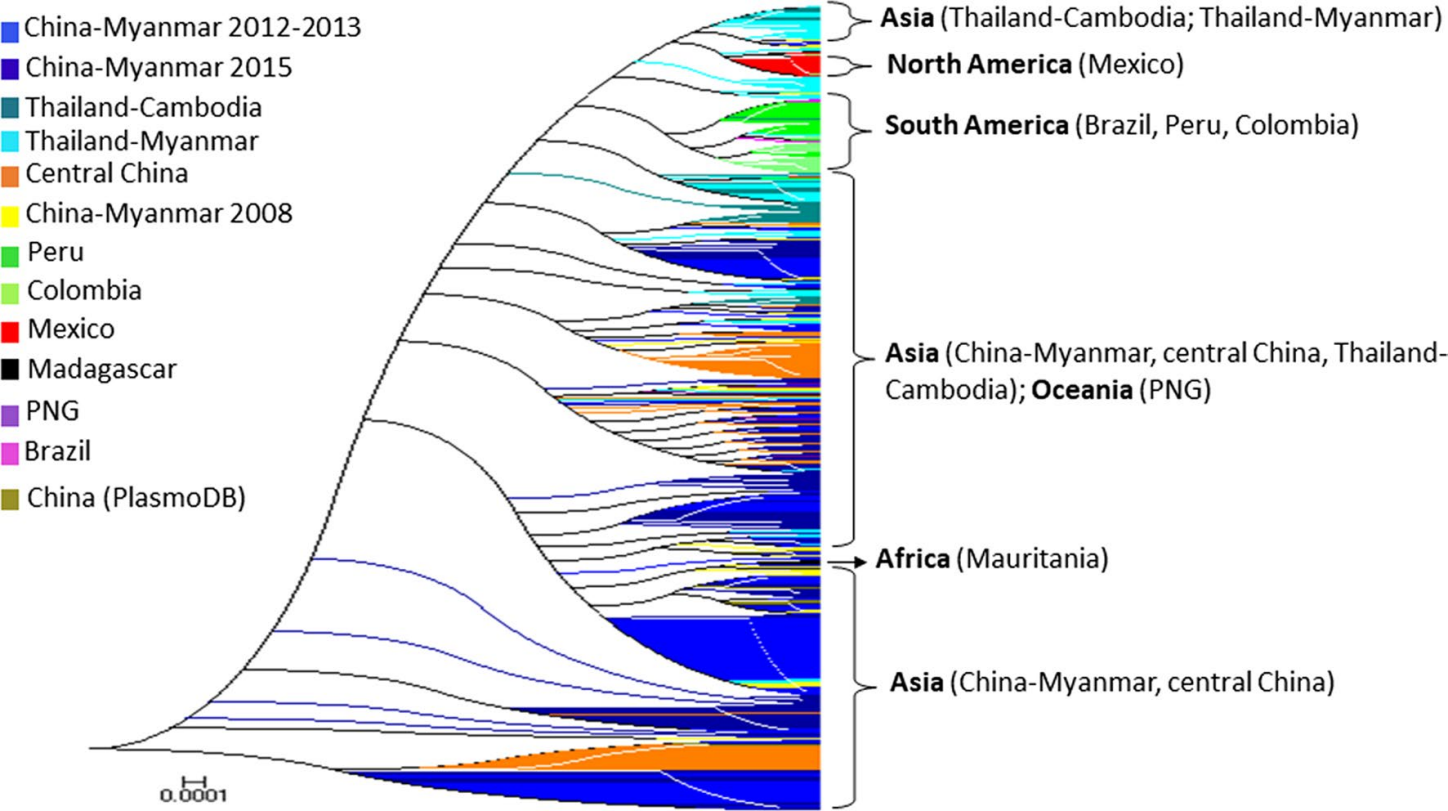
Phylogenetic analysis of *Pvmdr1* sequences from global *P. vivax* isolates. The genetic interrelationship between *P. vivax* parasites from the China–Myanmar border and global isolates was assessed using the Maximum Likelihood method with 1000 bootstraps

Based on the Pvmdr1 amino acid sequences, a total of 188 haplotypes were found in 510 parasites isolates from the world (Fig. 2). Most geographical regions except Madagascar and Mauritania had more than one predominant haplotype. There were significant differences in the number of haplotypes and prevalence of individual haplotypes among all the countries considered. The China–Myanmar border parasites had the highest number of 79 unique haplotypes, followed by parasites from Thailand (46 haplotypes). A minimum spanning network clearly showed geographical clustering of the haplotypes (Fig. 2). A continental, followed by a country-wise and then region-wise difference were observed. In this regard, the majorities of the Asian and African parasites are separated from the New World parasites (Fig. 3). Also, parasites from the Thailand–Myanmar border were distinctive from those from the Thailand–Cambodia border (Fig. 2). Similarly, temperate-zone *P. vivax* parasites from central China were completely separated from those of the China–Myanmar border. Only 20 haplotypes out of the 188 were shared across all populations in the world (Fig. 2). Besides, some long branches are present within the torso of the network (Fig. 2), highlighting a local genetic difference of the parasites. Significant sharing of the haplotypes was detected between physically connected Asian countries, suggesting potential genetic exchanges in the past between these populations. Interestingly, the African cluster was linked to one of the predominant haplotypes of the Asian cluster.

Phylogenetic analysis using the maximum likelihood method further corroborated the high-degree genetic differentiation of parasite populations from the three GMS border regions, as well as parasites from the rest of world (Fig. 3). The result showed a clear population substructure, in particular, within the China–Myanmar populations (Fig. 3).

Population differentiation examined through the estimation of *F*_ST_, the Wright’s fixation index of inter-population variance in allele frequencies, revealed large degrees of variation in population differentiation between countries (*F*_ST_ = 0.099–0.77), not considering India, Mauritania and North Korea due to very limited sample size. Overall, the *F*_ST_ estimate of the worldwide populations was 0.36, indicating that about 36% of the variation was apportioned between parasite populations. Great genetic differentiation was not only denoted between countries or regions, but also within some countries/regions such as the China–Myanmar border. Although there was a low degree of difference between parasites from the China–Myanmar border, the difference varied over the years (Table 5), with *F*_ST_ ranging from 0.025 to 0.105, suggesting extensive genetic inheritance. High degrees of genetic difference were detected among the South American countries (*F*_ST_ = 0.13–0.77, Table 5). Significant difference was also denoted between the Thailand–Myanmar border and the China–Myanmar border populations as well as between the Thailand–Myanmar border and the Thailand–Cambodia border populations (Table 5). Taken together, the result confirmed observations made in the phylogenetic and haplotype network analyses and is concordant with the hypothesis of the presence of genetic substructure.

**Table 5:** Pairwise *F*_ST_ estimates for worldwide *Plasmodium vivax* populations using *Pvmdr1* gene sequences

	Population	1	2	3	4	5	6	7	8	9	10	11	12
1	Central China 2004–2008												
2	CMB 2012–2013	0.290											
3	CMB 2015	0.129	0.085										
4	CMB 2008	0.284	0.025	0.105									
5	PNG	0.371	0.376	0.172	0.327								
6	China (PlasmoDB)	0.313	− 0.006	0.092	− 0.05	0.398							
7	Brazil	0.574	0.42	0.385	0.324	0.669	0.367						
8	Colombia	0.634	0.497	0.454	0.41	0.734	0.458	0.349					
9	Peru	0.563	0.403	0.366	0.299	0.658	0.348	0.126	0.226				
10	Mexico	0.661	0.524	0.467	0.438	0.774	0.492	0.415	0.499	0.381			
11	Thai-Myanmar	0.404	0.206	0.226	0.125	0.429	0.179	0.219	0.335	0.183	0.338		
12	Thai-Cambodian	0.370	0.373	0.298	0.279	0.492	0.376	0.327	0.432	0.283	0.453	0.168	
13	Madagascar	0.280	0.193	0.156	0.132	0.099	0.189	0.395	0.457	0.369	0.439	0.165	0.239

*Abbreviations*: CMB, China–Myanmar border; PNG, Papua New Guinea

**Fig. 4:**
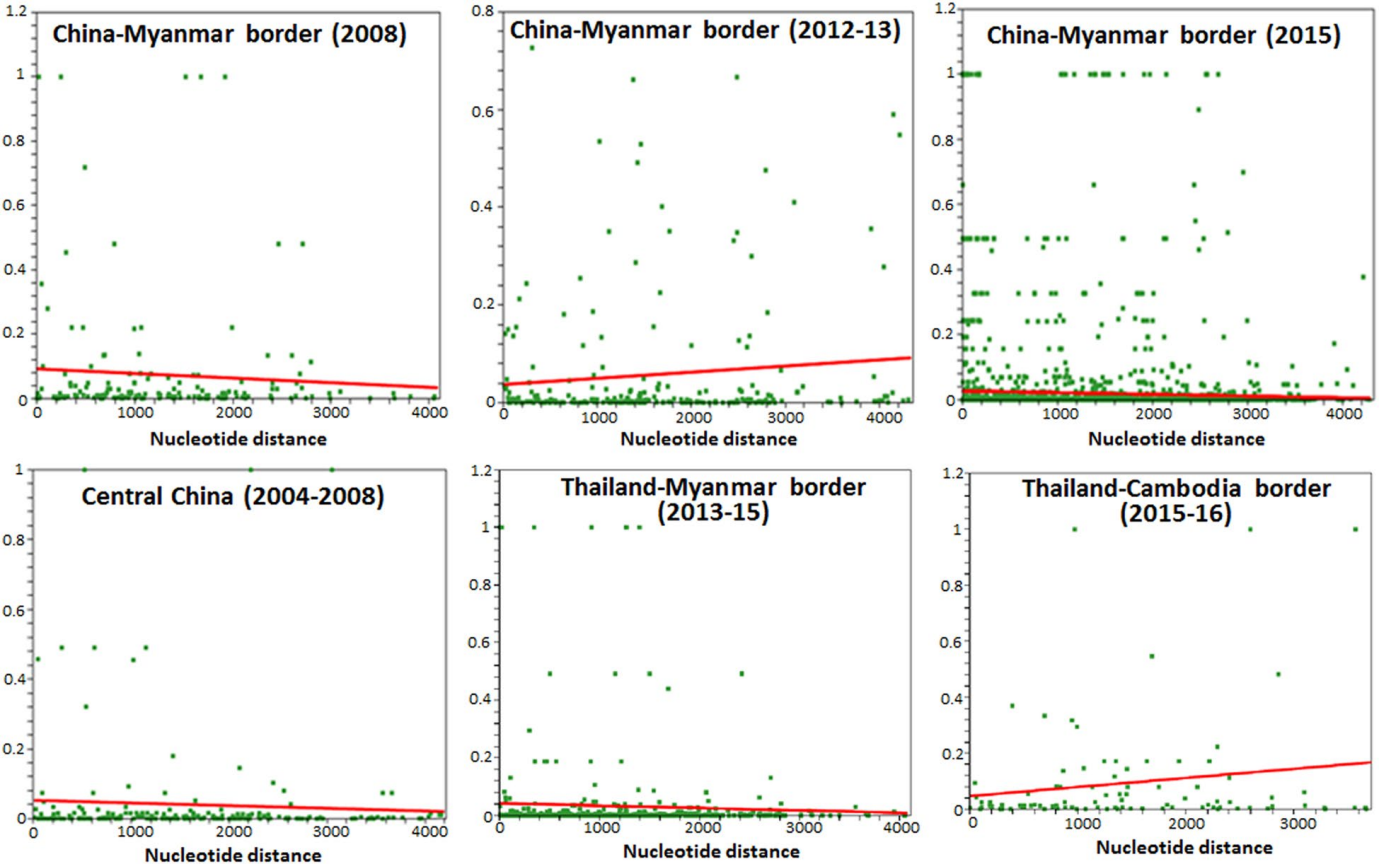
Linkage disequilibrium plots of *R*^2^ of the near-complete *Pvmdr1* gene in parasite populations from the GMS. Sites with significant linkage (*P* < 0.05) as calculated by Fisher’s exact test are shown as green squares above the red trace line, which represents the regression line

High LD was detected in the 2015 parasite population from the China–Myanmar border as compared to 2008 and 2012–2013 (Fig. 4), suggesting an effective population size reduction with the years. In contrast, limited LD was detected in central China, the Thailand–Cambodia and Thailand–Myanmar border populations (Fig. 4), suggesting an effective population expansion and isolation.

## Discussion

The GMS countries are aiming to eliminate malaria by 2030, and intensified malaria control efforts have led to a continuous decline of malaria incidence [58]. Malaria transmission is concentrated along international borders as these places are often mountainous, hard to reach, and inhabited by economically deprived populations [5, 6]. Some border areas had increased migrant populations as a result of political instability and military conflicts, leading to substantial changes in malaria epidemiology [59]. The Kachin civil wars resulted in the establishment of camps for internally displaced people in the China–Myanmar border area to host human population migrated from other malaria endemic areas. Human migration, together with the poor public health infrastructure, has led to a rising trend of malaria incidence and even malaria outbreaks in recent years [60]. Furthermore, cross-border human migration also poses another threat of malaria introduction to neighboring countries [6, 61]. Therefore, the China–Myanmar border region represents an interesting scenario for characterizing the parasite population structure and understanding their evolution during the course of malaria elimination.

This study focused on the molecular evolution of the *Pvmdr1* gene, a potential marker for CQ resistance, in order to address the deteriorating CQ efficacy at the China–Myanmar border areas [35]. The results showed an increase of the *Pvmdr1* genetic diversity at the China–Myanmar border over the years in spite of the intensified control measures in place. The genetic diversity of the *Pvmdr1* gene was high in most GMS border areas, though it fluctuated over the seven-year study period. As the frontline treatment for *P. vivax* malaria is CQ-PQ throughout the GMS, the genetic diversity of different parasite populations likely reflected the local differences in malaria epidemiology. Since most malaria endemic areas in the GMS also have sympatric *P. falciparum* transmission, different ACTs, especially the quinoline partner drugs, could have also exerted divergent selection pressures on the *Pvmdr1* gene. Moreover, this high genetic diversity might exemplify the impact of parasite introduction as found in preceding studies [61]. Three mutations (T958M, Y976F and F1076L) have been associated with *P. vivax* CQ resistance [23, 28, 30, 52]. The T958M mutation was fixed or approached fixation (98.3–100%) in the *P. vivax* populations from the China–Myanmar border as well as from the Thailand–Myanmar and Thailand–Cambodia borders, thus it is unlikely responsible for the reduced sensitivity of the parasites to CQ. However, the F1076L mutation in the longitudinal samples from the China–Myanmar border had an increasing trend in prevalence; its frequency reached 77.8% in 2015, almost doubled from that (41.7%) in 2012–2013. Interestingly, this mutation had a moderate prevalence in the Thailand–Myanmar border area (~30–62%) over the period of 2008–2016 and in Ubon Ratchathani (28%), but it almost reached fixation in Chanthaburi Province at the Thailand–Cambodia border [33, 62]. Conversely, the prevalence of the Y976F mutation in the China–Myanmar border populations progressively decreased and was not detected in the 2015 samples. Similarly, the Y976F prevalence also showed a decreasing trend in the Thailand–Myanmar and Thailand–Cambodia border samples collected in 2008 and 2014 [33, 62]. It is noteworthy that the Y976F mutation was associated with a low-level reduction of *in vitro* susceptibility to CQ [24, 25, 52]. It would be interesting to determine whether the reverse trends of the Y976F and F1076L mutations are associated with the decline of CQ efficacy in the GMS.

All the neutrality tests yielded negative values in most *P. vivax* populations, suggestive of the occurrence of rare alleles and parasites experiencing a directional selection or population expansion. Only the 2012–2013 parasites from the China–Myanmar border had positive values of those statistics, which may indicate a signature of balancing selection or population size decline [42, 43, 63, 64]. Inference of selection identified that the *Pvmdr1* gene has evolved under purifying selection, reflecting *Pvmdr1* as an essential gene. However, many tests that rely on differences between nonsynonymous and synonymous changes do not systematically take into account that positive selection often acts only on small regions of a gene product [65]. In fact, zooming in specific regions of Pvmdr1 also identified individual codons to be under positive selection in the studied parasite populations. Whereas F1076L was the only position found to be positively selected in Thailand–Cambodia border, several loci, including G698S, M908L and F1076L were under positive selection in the Thailand–Myanmar border and China–Myanmar border populations. This finding further corroborated an earlier analysis of the publicly available *P. vivax* genomes gathered from various sources, which similarly revealed that T958M and M908L, F1076L, G698S and S513R were under directional selection [66]. Of note, mapping the SNPs to the putative 3D model of PvMDR1 structure identified amino acid changes S513R, L845F, F1076L, K1393N and S1450L, all found to be under positive selection, could have impact on protein function [33, 52].

Both haplotype network and phylogenetic analysis revealed considerable clustering of the haplotypes relevant to the countries/continents of origin. This is intuitively understandable, as parasite populations in geographically separated continents or countries have been evolving under isolation, creating significant divergence among themselves. Under this scenario, parasites from Asia were more closely related among themselves than from those of the American origins, and parasites from the temperate region of central China were, to a large degree, separated from those of the tropical regions of the GMS. These comparisons also identified relatively little differentiation of parasite populations from the same geographical locations. Although the *Pvmdr1* genes from the GMS displayed high diversity, there was extensive sharing of the major haplotypes among these border parasite populations, suggesting little differentiation of parasite populations within the GMS. This observation was further reinforced by the very low *F*_ST_ value obtained among these GMS populations (*F*_ST_ = 0.025–0.085). Studies on vaccine candidate genes such as *PvAMA1* genes also showed high diversity and little differentiation of the *P. vivax* parasites from China–Myanmar border [67]. Nevertheless, parasite populations from the GMS did fall into several distinctive clades, suggesting the presence of gene flow barriers or/and divergent selection on the Pvmdr1 protein. This is plausible, as intensified control efforts of the malaria elimination campaign may have led to separated pockets of transmission hotspots, and these isolated parasite populations may have been evolving independently as what has been observed for the *P. falciparum* populations in the China–Myanmar border region [68]. This has also been the case of some genes such as the *P. falciparum* gamete surface protein gene *Pfs48/45* [69] and the *P. vivax* gamete surface protein gene *Pvs48/45* [70].

A temporal increase in LD was denoted in parasite populations from the China–Myanmar border. Central China as well as Thailand–Myanmar and Thailand–Cambodia borders had limited LD. This might suggest high level of inbreeding [7] and a history of bottleneck risen by an effective population size decline on the China–Myanmar border [71], whereas in the other borders and central China, there might be an expansion of the effective population size. Interestingly, despite observation of high numbers of minimal recombination events in the history of the China–Myanmar *P. vivax* populations, no recombination breakpoint was found in all of them. This latter finding reinforces our hypothesis of reduced effective population size resulting in high-level inbreeding and consequently strong LD on the China–Myanmar border. Frequent inbreeding and recombination between parasite genotypes also play a role in contributing to high genetic diversity within populations [72].

## Conclusions

Our study showed that the *Pvmdr1* gene in *P. vivax* populations of the China–Myanmar border area have undergone a strong diversification process with evidence of purifying selection on the whole gene and positive selection on certain loci of the gene. Furthermore, there was a low level of genetic differentiation among the GMS parasite populations, suggesting extensive gene flow within the GMS. The increased diversity of *P. vivax* found parasites from the China–Myanmar border suggests parasite introduction, which might be associated with the migration of human population from other *P. vivax* endemic regions of Myanmar due to military conflicts. The increase in LD with the years indicated expansion of particular parasite genotypes associated with the recent outbreaks of *P. vivax* malaria in recent years. These findings emphasize once more that effective management of clinical vivax cases and monitoring of human migration are indispensable for malaria elimination in the GMS.

## Supplementary information

**Additional file 1: Table S1.** Primers used for the amplification of the *Pvmdr1* gene.

**Additional file 2: Table S2.** All mutations with their domain localization and Provean and SIFT scores.

**Additional file 3: Table S3.** Mutations deleterious according to both types of software: Provean and SIFT.

## Data Availability

The datasets supporting the conclusions of this article are available the in additional files.

## Abbreviations

ABC: ATP-binding cassette
CQ: chloroquine
GMS: Greater Mekong Subregion
LD: linkage disequilibrium
MK: McDonald–Kreitman
NBD: nucleotide binding domain
PQ: primaquine
Pvmdr1: *Plasmodium vivax* multidrug resistance 1
SNP: single nucleotide polymorphism
TMD: transmembrane domain
