# Supplementary material for: Evolution of the Plasmodium vivax multidrug resistance 1 gene in the Greater Mekong Subregion during malaria elimination
Source: Parasit Vectors. 2020 Feb 12;13:67. doi: 10.1186/s13071-020-3934-5 (PMC7017538; doi:10.1186/s13071-020-3934-5)
Supplement: Supplementary file 1 — Additional file 1: Table S1. Primers used for the amplification of the Pvmdr1 gene. [file 13071_2020_3934_MOESM1_ESM.docx]

**Additional file 1: Table S1.** PCR primers used for amplification of the *Pvmdr1* gene

| **Primary PCR** | |
| --- | --- |
| P1F | AGCCTGCGCCCTCCCTTAC |
| P1R | CGTATATACATATGATCTGTG |
| **Semi-nest PCR** | |
| P1F | AGCCTGCGCCCTCCCTTAC |
| N-PR | GCATTGGTGTTACTTGCTTTG |
| N-PF | GACTCAGATATGAAGAGCAGC |
| P1R | CGTATATACATATGATCTGTG |
