# Supplementary material for: Evolution of the Plasmodium vivax multidrug resistance 1 gene in the Greater Mekong Subregion during malaria elimination
Source: Parasit Vectors. 2020 Feb 12;13:67. doi: 10.1186/s13071-020-3934-5 (PMC7017538; doi:10.1186/s13071-020-3934-5)
Supplement: Supplementary file 2 — Additional file 2: Table S2. All mutations with their domain localization and Provean and SIFT scores. [file 13071_2020_3934_MOESM2_ESM.docx]

Additional file 2: Table S2. All mutations with their domain localization and Provean and SIFT scores

| **AA mutation** | **Domain** | **PROVEAN score** | **Prediction (cutoff= -2.5)** | **SIFT Prediction** |
| --- | --- | --- | --- | --- |
| N13K |  | -0.426 | Neutral | Not tolerated |
| S192A | TM (3) | 1.394 | Neutral | Tolerated |
| N224I |  | -0.847 | Neutral | Tolerated |
| Y231D |  | -2.389 | Neutral | Tolerated |
| F290V | TM (5) | 1.602 | Neutral | Tolerated |
| V324G | TM (6) | -5.816 | Deleterious | Not tolerated |
| S334R | TM (6) | -2.360 | Neutral | Not tolerated |
| F336V | TM (6) | -3.283 | Deleterious | Tolerated |
| Y348D |  | -5.191 | Deleterious | Not tolerated |
| Y359D |  | -6.663 | Deleterious | Not tolerated |
| N371Y |  | -0.783 | Neutral | Tolerated |
| K376E |  | -1.710 | Neutral | Tolerated |
| I380L |  | -1.149 | Neutral | Tolerated |
| T409M |  | -1.233 | Neutral | Tolerated |
| K456T | AAA (1) | -2.840 | Deleterious | Not Tolerated |
| L470H | AAA (1) | -3.348 | Deleterious | Not Tolerated |
| S513R | AAA (1) | -0.331 | Neutral | Not Tolerated |
| G520D | AAA (1) | -0.130 | Neutral | Not Tolerated |
| V562G | AAA (1) | -6.732 | Deleterious | Not Tolerated |
| A593T | AAA (1) | -3.920 | Deleterious | Not Tolerated |
| I595F | AAA (1) | -3.320 | Deleterious | Not Tolerated |
| K599Q | AAA (1) | -2.332 | Neutral | Not Tolerated |
| L610F | AAA (1) | -3.920 | Deleterious | Not Tolerated |
| D611K | AAA (1) | -6.860 | Deleterious | Not Tolerated |
| N612T | AAA (1) | 1.584 | Neutral | Tolerated |
| K613Q | AAA (1) | 0.407 | Neutral | Tolerated |
| V618G | AAA (1) | -6.860 | Deleterious | Not Tolerated |
| K620E | AAA (1) | 1.145 | Neutral | Tolerated |
| T621K | AAA (1) | -1.480 | Neutral | Not Tolerated |
| N623I | AAA (1) | -4.323 | Deleterious | Not Tolerated |
| K672N |  | -0.141 | Neutral | Not Tolerated |
| G698S |  | -0.078 | Neutral | Tolerated |
| N740D |  | 0.668 | Neutral | Tolerated |
| A763V |  | 0.059 | Neutral | Tolerated |
| R803K |  | 0.102 | Neutral | Tolerated |
| P808T |  | -2.156 | Neutral | Tolerated |
| L838V | TM (7) | 0.911 | Neutral | Tolerated |
| F842V | TM (7) | -5.528 | Deleterious | Tolerated |
| L845F | TM (7) | -2.540 | Deleterious | Not Tolerated |
| A861E |  | 1.988 | Neutral | Tolerated |
| V872A | TM (8) | -1.334 | Neutral | Tolerated |
| N889H | TM (8) | -1.464 | Neutral | Not Tolerated |
| E896K |  | -0.400 | Neutral | Tolerated |
| M899L |  | 0.990 | Neutral | Tolerated |
| M908L |  | 1.221 | Neutral | Tolerated |
| E911K |  | -3.383 | Deleterious | Not Tolerated |
| S926A |  | -0.550 | Neutral | Tolerated |
| A927D |  | -3.070 | Deleterious | Tolerated |
| H928L |  | -1.378 | Neutral | Not Tolerated |
| D932N |  | -2.737 | Deleterious | Not Tolerated |
| L936F |  | -2.117 | Neutral | Not Tolerated |
| V945G | TM (9) | 1.910 | Neutral | Not Tolerated |
| I951K | TM (9) | -3.457 | Deleterious | Not Tolerated |
| V952L | TM (9) | -1.053 | Neutral | Tolerated |
| T958M | TM (9) | 1.270 | Neutral | Tolerated |
| V959G | TM (9) | -3.966 | Deleterious | Not Tolerated |
| Y976F | TM (10) | -0.337 | Neutral | Tolerated |
| F979S | TM (10) | 0.064 | Neutral | Tolerated |
| M980V | TM (10) | -0.141 | Neutral | Tolerated |
| E996Q |  | 0.771 | Neutral | Tolerated |
| K997R |  | -0.593 | Neutral | Tolerated |
| F1070V | TM (11) | -2.021 | Neutral | Tolerated |
| F1076L | TM (11) | -3.503 | Deleterious | Tolerated |
| P1177T |  | -6.472 | Deleterious | Not Tolerated |
| K1219E | AAA (2) | -0.011 | Neutral | Tolerated |
| H1222P | AAA (2) | -0.224 | Neutral | Tolerated |
| G1232C | AAA (2) | -0.595 | Neutral | Not Tolerated |
| G1265W | AAA (2) | -0.464 | Neutral | Not Tolerated |
| S1274R | AAA (2) | -2.543 | Deleterious | Tolerated |
| K1393N | AAA (2) | -3.507 | Deleterious | Not Tolerated |
| S1450L |  | -2.630 | Deleterious | Tolerated |
