# Supplementary material for: Evolution of the Plasmodium vivax multidrug resistance 1 gene in the Greater Mekong Subregion during malaria elimination
Source: Parasit Vectors. 2020 Feb 12;13:67. doi: 10.1186/s13071-020-3934-5 (PMC7017538; doi:10.1186/s13071-020-3934-5)
Supplement: Supplementary file 3 — Additional file 3: Table S3. Mutations deleterious according to both types of software: Provean and SIFT. [file 13071_2020_3934_MOESM3_ESM.docx]

Additional file 3: Table S3. Mutations deleterious according to both types of software: Provean and SIFT

| **AA mutation** | **Domain** | **PROVEAN score** | **Prediction (cutoff= -2.5)** | **SIFT Prediction** |
| --- | --- | --- | --- | --- |
| V324G | TM (6) | -5.816 | Deleterious | Not tolerated |
| Y348D |  | -5.191 | Deleterious | Not tolerated |
| Y359D |  | -6.663 | Deleterious | Not tolerated |
| K456T | AAA (1) | -2.840 | Deleterious | Not Tolerated |
| L470H | AAA (1) | -3.348 | Deleterious | Not Tolerated |
| V562G | AAA (1) | -6.732 | Deleterious | Not Tolerated |
| A593T | AAA (1) | -3.920 | Deleterious | Not Tolerated |
| I595F | AAA (1) | -3.320 | Deleterious | Not Tolerated |
| L610F | AAA (1) | -3.920 | Deleterious | Not Tolerated |
| D611K | AAA (1) | -6.860 | Deleterious | Not Tolerated |
| V618G | AAA (1) | -6.860 | Deleterious | Not Tolerated |
| N623I | AAA (1) | -4.323 | Deleterious | Not Tolerated |
| L845F | TM (7) | -2.540 | Deleterious | Not Tolerated |
| E911K |  | -3.383 | Deleterious | Not Tolerated |
| D932N |  | -2.737 | Deleterious | Not Tolerated |
| I951K | TM (9) | -3.457 | Deleterious | Not Tolerated |
| V959G | TM (9) | -3.966 | Deleterious | Not Tolerated |
| P1177T |  | -6.472 | Deleterious | Not Tolerated |
| K1393N | AAA (2) | -3.507 | Deleterious | Not Tolerated |
